# Spectral Response of an Embedded Fiber Bragg Grating to an Arbitrary Non-Axial Mechanical Impact

**DOI:** 10.3390/s26206621

**Published:** 2026-10-19

**Authors:** Dmitry Arseniev, Dmitry Kiesewetter, Sergey Krivosheev, Sergey Magazinov, Victor Malyugin, Ivan Kuznetsov, Sergey Varzhel, Andrei Anatolevich Dmitriev, Victoriia Klishina, Andrei Danilov, Anastasiya Magomedova, Leonid Sverdlik, Xue Han, Changsen Sun

**Affiliations:** 1 Higher School of High Voltage Engineering, Institute of Energy, Peter the Great St. Petersburg Polytechnic University, St. Petersburg 195251, Russia; vicerector.int@spbstu.ru (D.A.); ksi.mgd@gmail.com (S.K.); magazinov_sg@mail.ru (S.M.); vim@spbstu.ru (V.M.); kuznetsov_ib@inbox.ru (I.K.); 2 Scientific Research Center of Light-Guiding Photonics, ITMO University, Kronverksky Pr. 49, bldg. A, St. Petersburg 197101, Russia; svvarzhel@itmo.ru (S.V.); dmitrievaa1994@yandex.ru (A.A.D.); novivial@mail.ru (V.K.); aydanilov@itmo.ru (A.D.); aamagomedova@itmo.ru (A.M.); 3 High-Altitude Observatory of Atmospheric Physics, Kyrgyz-Russian Slavic University, Bishkek 720000, Kyrgyzstan; l.sverdlik@mail.ru; 4 College of Optoelectronic Engineering and Instrumentation Science, Dalian University of Technology, Dalian 116024, China; xue_han@dlut.edu.cn (X.H.); suncs@dlut.edu.cn (C.S.)

**Keywords:** fiber Bragg grating, mechanical stress, deformation, non-axial loading, spectral response, pulse action, measurements, numerical simulation

## Abstract

In most cases, the properties of fiber Bragg gratings (FBGs) are considered under axial tension or compression. The effects caused by induced anisotropy of the refractive index in FBGs under the action of a transverse load are also known. However, the change in the radiation spectrum reflected from FBGs under the influence of a load acting at an arbitrary angle to the FBG axis on the grating embedded in the elements of the mechanical system has not been studied in sufficient detail. This paper theoretically and experimentally investigated the change in the resonant wavelength of FBGs attached to a disk under the action of a compressive force acting at an arbitrary angle to the FBG axis. The FBG deformation in the static loading mode is theoretically estimated and compared with the experiment. The angle between the direction of the force applied and the FBG axis is determined at which the compression deformation turns into a tensile deformation is determined. It is shown that there is a good agreement between the theoretical and experimental data. It is established that the studied effects for the static load mode also occur under pulsed mechanical load, but with some specifics. The effects described make it possible to more accurately calculate the response of an embedded FBG to mechanical impact.

## 1. Introduction

Fiber Bragg gratings (FBGs) are widely use in engineering as a temperature and deformation sensors. The principle of operation and basic properties of FBG are summarized in many reviews of the literature, for example, in [1,2,3,4,5]. Some properties of embedded FBGs and systems for the diagnosis of high-speed deformations are presented, in particular, in [6,7,8,9,10]. The most common case is axial (longitudinal) stretching or compression of the FBG. In this case, the displacement of the resonant wavelength Δλ of a fiber Bragg grating can be described the following formula [1,2,3,4,5,11,12]:

(1) $$\Delta \lambda =\lambda_{B0}\left(1-p_{e}\right)\epsilon$$

where $\lambda_{B0}$ is the resonant wavelength of the FBG in the absence of deformation, $p_{e}$ is the effective elastic-optical constant and ϵ is the relative deformation. For a given wavelength $\lambda_{B0}$ at $\Delta \lambda \ll \lambda_{B0}$, Expression (1) can be written as:

(2) $$\Delta \lambda =k_{B}\epsilon$$

where $k_{B}$ is the proportionality coefficient. For a standard single-mode optical fiber at a wavelength of 1550 nm, the $k_{B}$ coefficient is approximately 12 nm per % or $1.2\times 10^{-3}$ nm per μϵ. If the optical parameters of the fiber material and its waveguide properties did not change during stretching or compression, then the relative displacement of the resonant wavelength of the FBG would correspond to the magnitude of the relative deformation ($\Delta \lambda /\lambda_{B0}=\epsilon$). That is, $k_{B}/\lambda_{B0}$ would be equal to one. The presence of these effects, primarily the elastic-optical effect, leads to the fact that there is a $k_{B}/\lambda_{B0}\approx 0.78$, i.e., the elastic-optical effect reduces the effect of deformation on the displacement of the wavelength Δλ.

A transverse compressive force can act on the FBG, leading to a change in the refractive index of the FBG material, which leads to birefringence in the optical fiber [11] and a shift in the resonant wavelength of the FBG. Several works are devoted to the consideration of these effects, in particular, in [2,3,12,13,14]. A detailed theoretical consideration of this effect is given, for example, in [14], where, among other things, it is shown that the dependence of the displacement of the resonant wavelength of the FBG on the applied lateral force depends on the polarization of the radiation and may have different signs of the derivative (i.e., Δλ is greater than and less than zero) in the case of plane strain. Also in [14], an example of splitting the reflection spectrum of a distributed FBG in the case of plane strain and two different applied force values is given.

A theoretical consideration of the effect of non-axial load on the reflection spectrum of the FBG is also given in [13], where it is shown that peak separation is proportional to the difference between the transverse strains $\epsilon_{x}$ and $\epsilon_{y}$ only and a formula is given for calculating the shift difference of the resonant wavelengths $\Delta \lambda_{B,y}$ and $\Delta \lambda_{B,x}$:

(3) $$\frac{\Delta \lambda_{B,y}-\Delta \lambda_{B,x}}{\lambda_{B0}}=\frac{n_{eff}}{2}\left(p_{12}-p_{11}\right)\left(\epsilon_{y}-\epsilon_{x}\right)$$

where $\epsilon_{x}$, $\epsilon_{y}$ are the relative deformation along the OX and OY axes, $\lambda_{B0}$ is the resonant wavelength of the FBG in the absence of FBG deformation, $n_{eff}$ is the effective refractive index of the core of the unstressed fiber; $p_{11}$ and $p_{12}$ are the Pockel’s strain-optic coefficients

Splitting of the peak under lateral load on the FBG is also considered in [15], the theory and numerical modeling of the FBG spectra under transverse load in [16]. A study of fiber Bragg gratings spectral shaping when a transverse force perturbs a small grating section is presented in [17]. The evolution of the reflection spectrum from single mode (SM) FBG subject to a local transverse load and subject to a constant pressure with temperature in [18] was theoretically and experimentally determined. The behavior of fiber Bragg grating sensors subjected to transversal as well as axial strains, in particular, for high-birefringent and low-birefringent polarization-maintaining fiber is considered [19,20,21]. It was shown in [21] that the spectrum of reflected radiation from a transversely loaded FBG axis contains two peaks corresponding to two orthogonal modes of fiber polarization, and if the axial deformation and temperature in the fiber are known, then two components of the transverse deformation can be calculated from changes in the wavelength of the two peaks. The specific effect of changing the reflection spectrum of the FBG is the failure of Transmission Dips at 2/3 of the Bragg Wavelength, under transverse mechanical load, which is considered in [22].

Thus, when considering the effect of three-dimensional deformations on the value of Δλ for FBGs attached to elements of mechanical structures located inside the medium under study, etc., i.e., mechanical influences at an arbitrary angle relative to the axis of the aircraft, the effect of transverse mechanical load and the mechanical properties of the material to which the FBG is connected should be taken into account. However, the effect of mechanical action at an arbitrary angle to the FBG axis on the reflection spectrum, as well as under pulsed mechanical action, has not been studied before.

This work is devoted to the consideration of one of the cases, the study of the mechanical static and pulsed effects on a FBG glued to a polymer disk in an arbitrary direction of action relative to the axis of the FBG.

## 2. Basic Theoretical Concepts

The problem of calculating the mechanical stress σ in a disk when two equal and opposite forces *F* acting along the diameter have an analytical solution, which is given, in particular, in [23]. Let force *F* be applied along the OY axis. In the center of the disk, the mechanical stress along the OY and OX axes can be determined by the following formulas [23]:

(4) $$\sigma_{y}=-\frac{6F}{\pi dh}\;$$

(5) $$\sigma_{x}=\frac{2F}{\pi dh}\;$$

where *d* and *h* are the diameter and thickness of the disk.

In particular, the mechanical stress along the OY axis was 63.6 and 127.3 kPa, and along the OX axis, it was 21.2 and 42.4 kPa, respectively, for the disk used in the experiment, with an applied acting force of 50 N and 100 N. Numerical modeling performed using the finite element method confirmed the correctness of the analytical expressions.

Then, the normal strain in *x* and *y* direction, taking into account the Poisson’s ratio μ and the Young’s modulus *E*, can be calculated using the following formulas [23]:

(6) $$\epsilon_{y}=-\left(\sigma_{y}-\mu \sigma_{x}\right)/E=-\frac{2F}{E\pi dh}\left(3+\mu \right)$$

(7) $$\epsilon_{x}=\left(\sigma_{x}-\mu \sigma_{y}\right)/E=\frac{2F}{E\pi dh}\left(1+3\mu \right)$$

In particular, from Formulas (6) and (7) it follows that

(8) $$\frac{\epsilon_{y}}{\epsilon_{x}}=-\frac{3+\mu }{1+3\mu }$$

For polymethylmethacrylate (PMMA) with a Poisson’s ratio of μ≈0.35, the ratio $\epsilon_{x}/\epsilon_{y}$ is approximately equal to −0.612. Let us introduce normalized values of relative deformation by dividing Expressions (6) and (7) by 2F/(Eπdh):

(9) $$\epsilon_{ny}=-3-\mu$$

(10) $$\epsilon_{nx}=1+3\mu$$

This will simplify the consideration of the dependence on the angle α and will allow us to compare the magnitude of the measured force *F* and the change in the resonant wavelength FBG, which have different units of measurement. Normalization to values at α=0 also makes it possible to exclude the dependence of the applied force.

The longitudinal $\sigma_{l}$ and transverse $\sigma_{p}$ mechanical stresses, as well as the relative deformation of $\epsilon_{l}$ and $\epsilon_{p}$ along the FBG axis located at an angle α to the OY axis, are determined by the following expressions [23]:

(11) $$\sigma_{l}\left(\alpha \right)=\sigma_{x}\sin^{2}\left(\alpha \right)+\sigma_{y}\cos^{2}\left(\alpha \right)$$

(12) $$\sigma_{p}\left(\alpha \right)=\sigma_{x}\cos^{2}\left(\alpha \right)+\sigma_{y}\sin^{2}\left(\alpha \right)$$

(13) $$\epsilon_{l}=\left(\sigma_{l}\left(\alpha \right)-\mu \sigma_{p}\left(\alpha \right)\right)/E$$

(14) $$\epsilon_{p}=\left(\sigma_{p}\left(\alpha \right)-\mu \sigma_{l}\left(\alpha \right)\right)/E$$

The magnitude of the normalized longitudinal $\epsilon_{nl}$ and transverse $\epsilon_{np}$ deformations as a function of angle α can be represented as:

(15) $$\epsilon_{nl}\left(\alpha \right)=\left(1+3\mu \right)\sin^{2}\left(\alpha \right)-\left(3+\mu \right)\cos^{2}\left(\alpha \right)$$

(16) $$\epsilon_{np}\left(\alpha \right)=\left(1+3\mu \right)\cos^{2}\left(\alpha \right)-\left(3+\mu \right)\sin^{2}\left(\alpha \right)$$

Expressions (15) and (16) can be converted to the form:

(17) $$\epsilon_{nl}\left(\alpha \right)=\mu \left(1-2\cos\left(2\alpha \right)\right)-\left(1+2\cos\left(2\alpha \right)\right)$$

(18) $$\epsilon_{np}\left(\alpha \right)=\mu \left(1+2\cos\left(2\alpha \right)\right)-\left(1-2\cos\left(2\alpha \right)\right)$$

The change in the resonant wavelength of the FBG is mainly determined by the longitudinal component of the deformation, and the transverse deformation can affect the change in $\lambda_{B0}$ through a change in the refractive index of the fiber core. We will determine the change in longitudinal deformation, and then the change in wavelength relative to the initial point of the angular dependence α = 0, setting this ratio to −1. Then:

(19) $$\frac{\epsilon_{nl}\left(\alpha \right)}{-\epsilon_{nl}\left(\alpha =0\right)}=\frac{\left(1+3\mu \right)\sin^{2}\left(\alpha \right)-\left(3+\mu \right)\cos^{2}\left(\alpha \right)}{3+\mu }$$

or

(20) $$\frac{\epsilon_{nl}\left(\alpha \right)}{-\epsilon_{nl}\left(\alpha =0\right)}=\frac{\mu (1-2\cos(2\alpha ))-(1+2\cos(2\alpha ))}{3+\mu }$$

## 3. Measurement Methods

**Figure 1:**
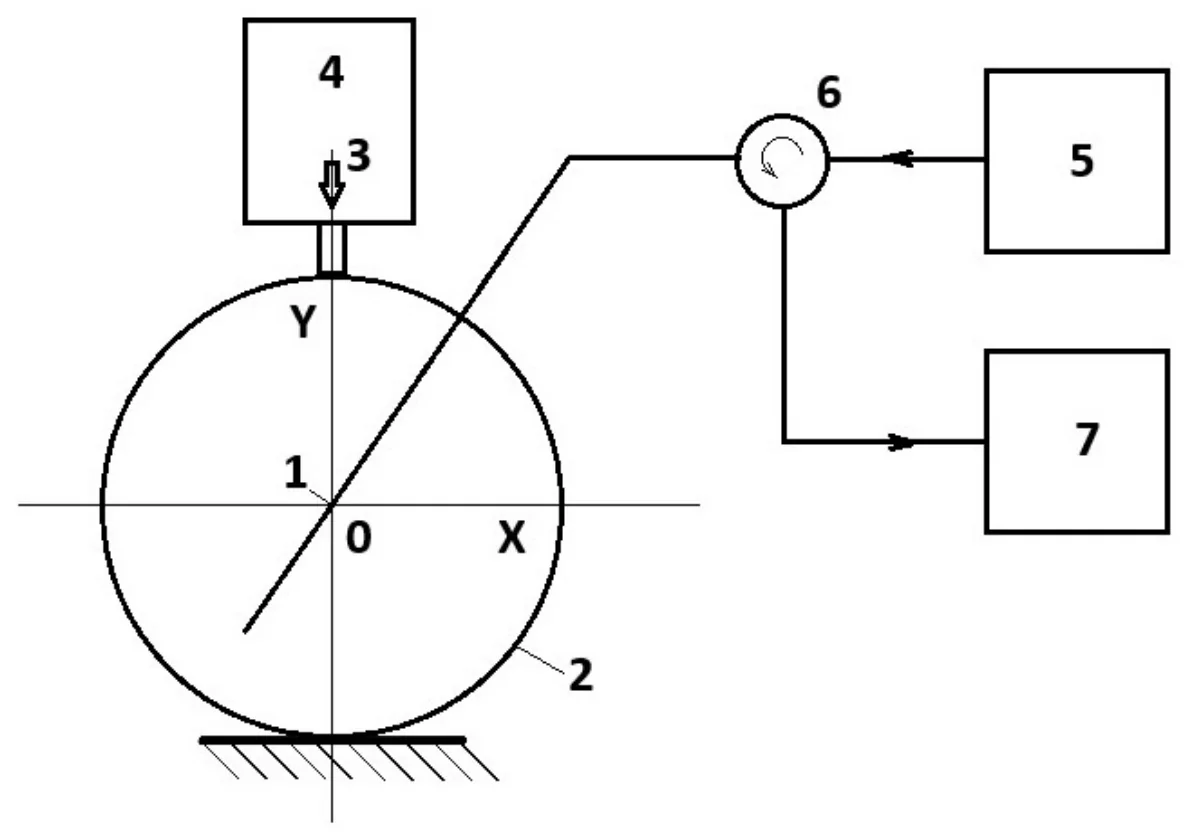
Experimental setup scheme: 1 is the fiber Bragg grating, 2 is the PMMA disc, 3 is the direction of compression of the disc by press, 6 is the circulator; for static load: 4 is the dynamometer, 5 is the superluminescent diode, 7 is the the spectroanalyzer; for pulsed mechanical load: 4 is the tube, 5 is the semiconductor laser, 7 is the photodetector.

The study of the effect of a non-axial load on the change in the parameters of reflected FBG radiation was performed under both static and pulsed mechanical loads. The object of this study was FBG 1 (Figure 1), glued to a disk 2 made of polymethylmethacrylate (PMMA) (Magatakt Co Ltd, Russia) with a diameter of 0.25 m and a thickness of 6 mm. Ethyl cyanoacrylate (ECA) (LAB Industries, Russia) glue was used, and the thickness of the adhesive layer between the optical fiber with FBG and the PMMA surface did not exceed 100 μm. The length of the FBG was 5 mm. In the general case, the calculation of the deformation of the embedded FBG through the protective shells and the adhesive layer based on the deformation of the surrounding FBG environment presents a serious difficulty [24]. But in this work, the optical fiber with FBG has been stripped of its protective shells. This suggests that the deformation of the FBG and PMMA discs in the area of adhesive contact is approximately the same [25]. The shape of the reflection spectrum of the FBG was close to Gaussian with a width of 0.23 nm at the 3 dB level and a reflection coefficient of approximately 50%. The FBG was obtained using the inscription scheme presented in [26,27].

The scheme of the experimental setup is shown in Figure 1.

The pressure on the disk was applied by press 3 to the end surface of the disk and the impact force was measured by dynamometer 4 attached to the movable part of the press. The FBG disk was vertically positioned between the base of the press and the tip of the dynamometer. The FBG center was located in the center of the disk. The angle α between the applied force direction and the FBG axis could be changed by turning the disk. The accuracy of measuring the force was ±1%, the angle was $\pm 1^{\circ }$. In the work, standard optical fibers SMF-28 (G.652), fiber-optic components that do not support the polarization of propagating radiation (FBG, optical fibers, circulator) and polarization insensitive devices (light source, spectrometer) were used. A superluminescent diode 5 was used as an optical radiation source, the radiation from which was fed through a circulator 6 into a fiber optic fiber with FBG. The spectrum of reflected radiation was recorded by an Anritsu MS9740B optical spectrum analyzer 7. During the measurements, the ambient temperature, respectively, of the disk and FBG, could be considered constant.

The size and shape of the sample on which the FBG is pasted were chosen for the following reasons. The round shape of the sample makes it possible to provide the same mechanical stress in the area of the glued FBG at an arbitrary angle of the FBG axis relative to the direction in which the force is applied. The small diameter of the disk does not allow for uniform mechanical stress in the FBG region; with a large one, considerable effort is required to achieve a relatively small FBG deformation. When a thin disk is compressed, it can bend, which will lead to a distortion of the studied dependencies. With a large thickness of the disk, significant compressive force must be applied, which can lead to nonlinear effects in areas with high mechanical stress (near the points in which the forces are applied).

As shown by numerical modeling and experimental investigation, the results of which are given below, the change in the resonant wavelength of FBG at maximum load (200 N) was no more than 0.1 nm at wavelength of 1310 nm. Taking into account that the temperature shift of the FBG resonant wavelength in a standard fiber is more than 0.01 nm per ◦C, for acceptable measurement accuracy, it is desirable to ensure temperature stability during measurements with an accuracy of $0.1^{\circ }$C.

To study the operation of FBG under pulsed mechanical influences, the press was replaced with a mechanical disk mounting system, and the dynamometer was replaced with a metal tube. The pulse effect was created by the impact of a falling metal cylinder with a diameter of 6 mm from a height of 0.15 m passing through the tube and falling on the end of the disk. In this case, the semiconductor laser mounted on a Peltier element was used as the radiation source. The wavelength of the $\lambda_{LD}$ laser was approximately 1310 nm with a half-width of $\sigma_{LD}=0.015$ nm. The laser wavelength was adjusted by changing the laser temperature. The wavelength $\lambda_{LD}$ of the laser radiation was chosen so that the reflected radiation power was 1/2 of the maximum value, which approximately corresponded to the inflection point of the Gaussian function—$\lambda_{B}\pm \sigma_{s}/2^{1/2}$. This provided high sensitivity and, as previously shown, a close to linear response to mechanical action [28].

When disk 2 was rotated, the wavelength of the SL radiation was also adjusted to compensate for the change in the semiconductor laser wavelength under the influence of gravity acting on the mass of the disk. The displacement of the resonant wavelength FBG under pulsed mechanical action was determined by the change in the magnitude of the radiation reflected from FBG according to the Formula (24). To reduce the effect of temperature on the measurement results, measurements were performed initially under a mechanical load of 50 N, and then, after the load was removed, after the shortest possible delay.

Application of FBG with Gaussian reflection spectrum:

(21) $$s\left(\lambda \right)=\frac{p_{0}}{\sqrt{\pi }\sigma_{s}}k_{r}\exp\left(-\frac{\Delta \lambda^{2}}{\sigma_{s}^{2}}\right)$$

where *s* is the spectral density, $p_{0}$ is the power of the radiation incident on FBG, $k_{r}$ is the reflection coefficient of FBG, Δλ is the displacement relative to the resonant wavelength FBG, $\sigma_{s}={\left(\sigma_{LD}^{2}+\sigma_{FBG}^{2}\right)}^{1/2}$ is the equivalent half-width of the reflection spectrum, $\sigma_{LD}$, and $\sigma_{FBG}$ are the half-width of the semiconductor laser (SL) radiation spectrum and the reflection spectrum FBG, has made it easier to determine the displacement of the resonant wavelength FBG under the action of deformation [28].

It is advisable to choose the operating wavelength $\lambda_{w}$ of the SL such that the reflected power is close to 1/2 [25]. Let $\Delta \lambda_{w}=\lambda_{b}-\lambda_{w}$ be the displacement of the wavelength at the operating point relative to the resonant wavelength. If, under the influence of deformation, the resonant wavelength FBG has changed by the value $\Delta \lambda_{\epsilon }$, then Formula (2) becomes as follows:

(22) $$U_{ph}=U_{m}\exp\left(-{\left(\frac{\Delta \lambda_{w}+\Delta \lambda_{\epsilon }}{\sigma_{s}}\right)}^{2}\right)$$

The dependence of the change in the output voltage $U_{ph}$ at the output of the photodetector on $\Delta \lambda_{\epsilon }$ and vice versa—$\Delta \lambda_{\epsilon }$ on $U_{ph}$ can be calculated using the Formula (22) if the value of $U_{ph}$ is known at $\Delta \lambda_{w}=0$ and $\Delta \lambda_{b}$. For a relatively small displacement of $\Delta \lambda_{\epsilon }$, the linear approximation $U_{ph}\left(\Delta \lambda_{b}\right)$ can be used. For the general case, we can write:

(23) $$\frac{dU}{\Delta \lambda_{\epsilon }}{|}_{\begin{array}{c} \Delta \lambda_{\epsilon }=0 \end{array}}=-\frac{2U_{m}\Delta \lambda_{w}}{\sigma_{s}^{2}}\exp\left(-\frac{\Delta \lambda_{w}^{2}}{\sigma_{s}^{2}}\right)$$

Accordingly:

(24) $$\Delta U_{ph}=-\frac{2U_{m}\Delta \lambda_{w}}{\sigma_{s}^{2}}\exp\left(-\frac{\Delta \lambda_{w}^{2}}{\sigma_{s}^{2}}\right)\Delta \lambda_{\epsilon }$$

In normalized form, Formula (24) has the form:

(25) $$\frac{\Delta U_{ph}}{U_{ph}\left(\lambda_{w}\right)}=-\frac{2\Delta \lambda_{w}}{\sigma_{s}^{2}}\Delta \lambda_{\epsilon }$$

For the dependencies discussed below, it is only important that $\Delta U_{ph}\propto \Delta \lambda_{\epsilon }$ for $U_{ph}=const$.

## 4. Data Obtained

**Figure 2:**
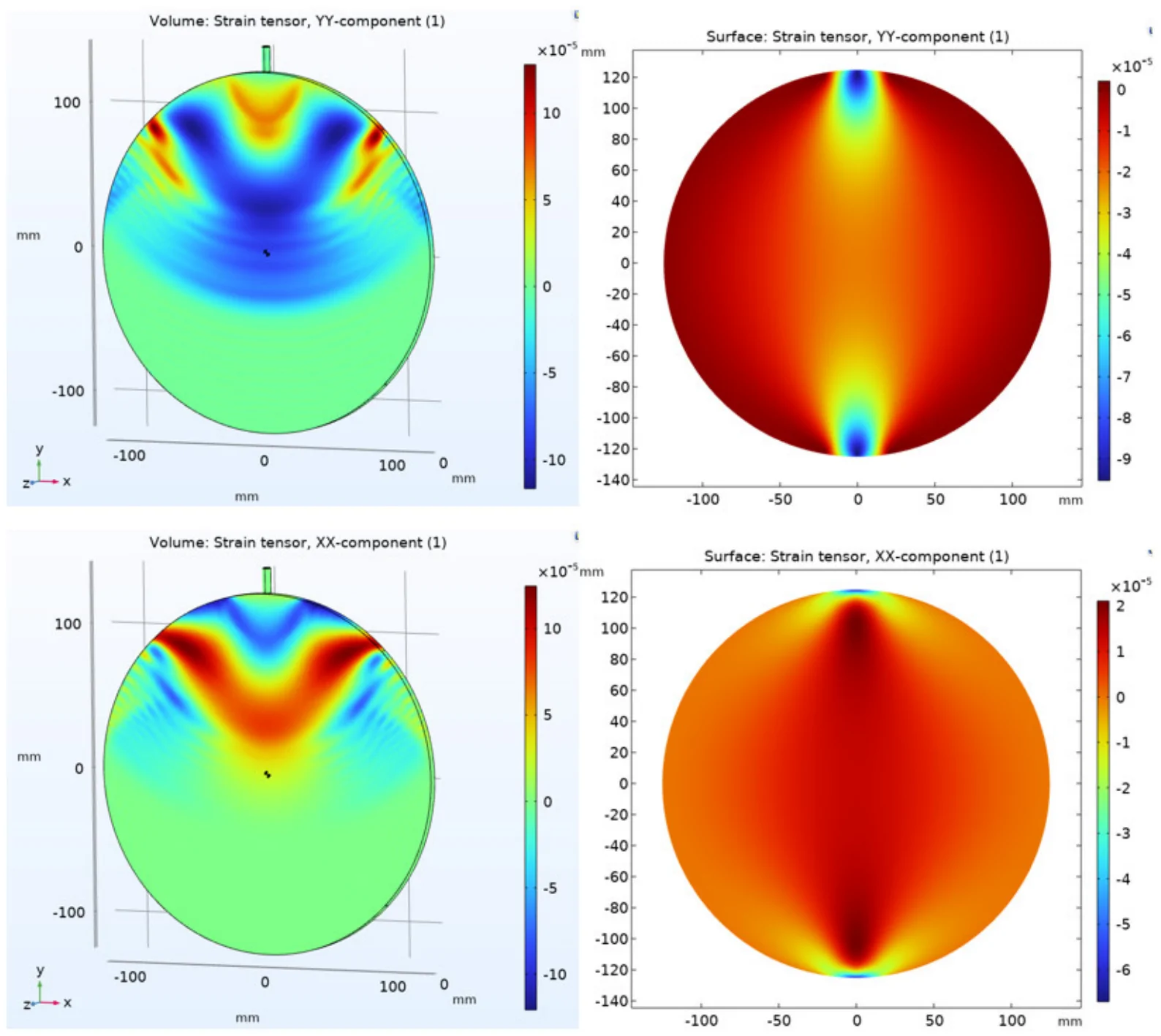
An example of components distribution of the strain tensor in static and dynamic mode.

The numerical simulation of the test was performed using the finite element method in COMSOL (Vers. 6.2). An example of the components distribution of the strain tensor along the longitudinal OY and transverse directions OX are shown in Figure 2.

**Figure 3:**
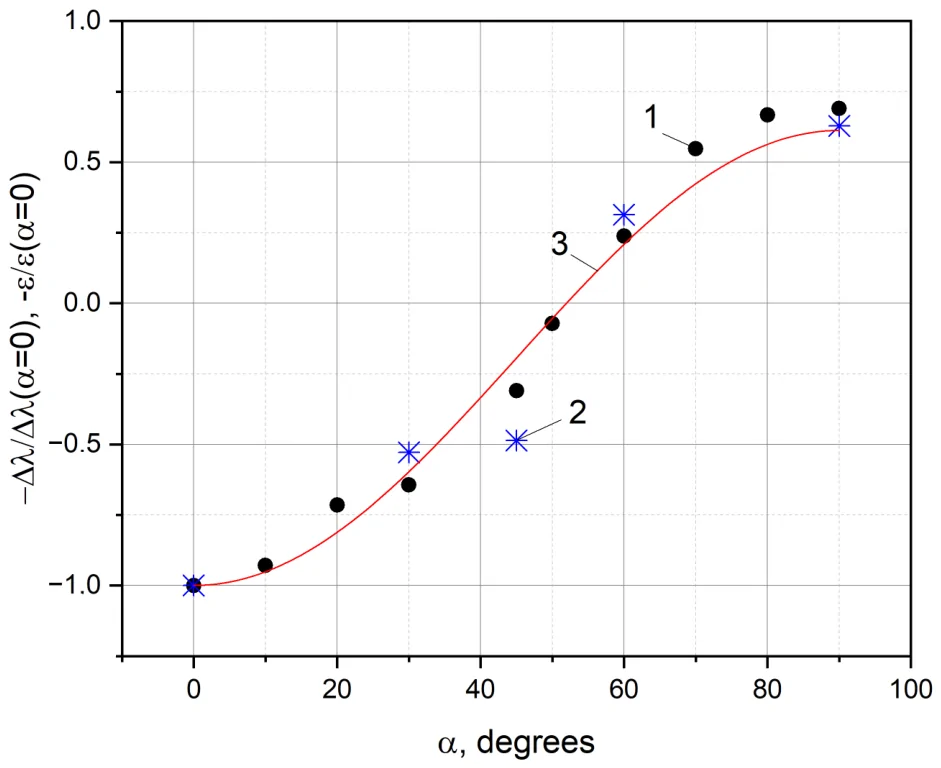
The dependence of the displacement of the resonant wavelength FBG on the angle of inclination FBG α at a constant value of the compressive force: 1—experiment; measurements using the spectrometer at F=100 N, 2—experiment; measurements by changing the power of reflected radiation at F=50 N, 3—calculation by Formula (19).

Experimentally, the dependence of the displacement of the resonant wavelength FBG Δλ on the angle α between the FBG axis and the direction of application of the compressive force (F=const), as well as the dependence Δλ on the magnitude of the compressive force at various angles α, was measured. The dependence obtained −Δλ(α)/Δλ(α=0) on F=100 N is shown in Figure 3. Figure 3 also shows the theoretical dependence $-\epsilon_{nl}\left(\alpha \right)/\epsilon_{nl}\left(\alpha =0\right)$, obtained using Formula (19) and the measurement result Δλ, based on the measured change in the radiation power reflected from FBG.

It should be noted that there is a good agreement between the theoretical and experimentally measured values of $\epsilon_{nl}\left(\alpha =90^{\circ }\right)/\epsilon_{nl}\left(\alpha =0\right)$ and $\Delta \lambda \left(\alpha =90^{\circ }\right)/\Delta \lambda \left(\alpha =0\right)$ theoretical −0.612, experimentally obtained −0.69 and −0.63 (see Figure 3). The angle $\alpha_{0}$, at which the dependence $\epsilon_{nl}\left(\alpha \right)/\epsilon_{nl}\left(\alpha =0\right)$, in accordance with Formulas (19) and (20), reaches zero can be calculated analytically:

(26) $$\alpha_{0}=\frac{1}{2}\arccos\left(\frac{1}{2}\cdot \frac{\mu -1}{\mu +1}\right)$$

and approximately equal to $51.96^{\circ }$. According to the measurement results shown in Figure 3, the specified angle is in the range from $50^{\circ }$ to $60^{\circ }$. In particular, when approximating dependence 1 by in the third degree polynomial, the specified angle is approximately $52.1^{\circ }$, and dependence 2 by the second degree polynomial is $57.9^{\circ }$.

**Figure 4:**
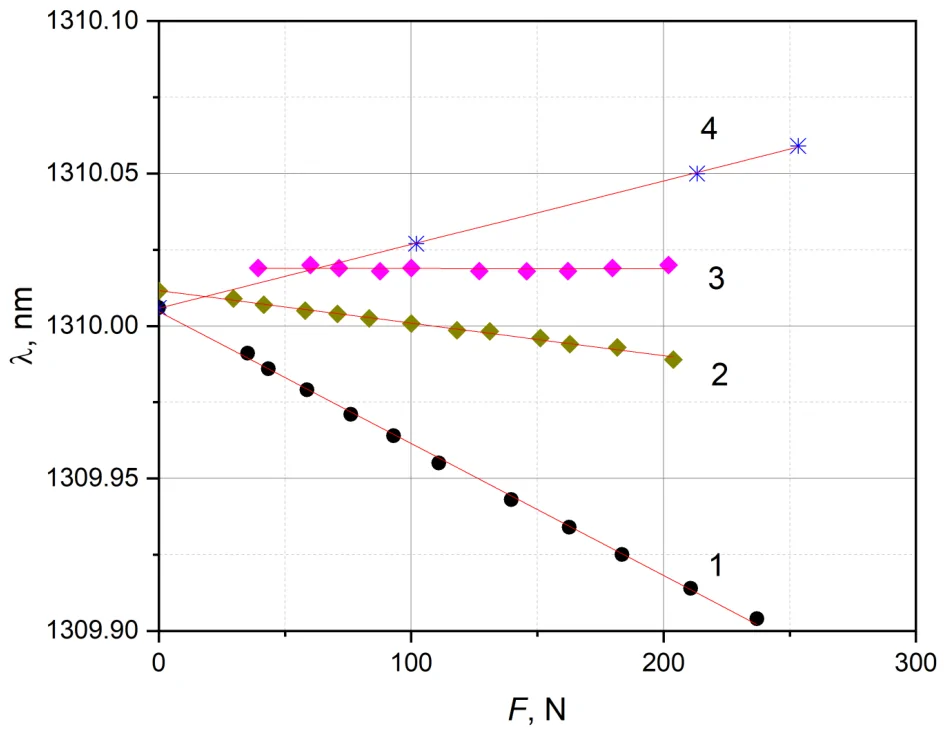
An example of the dependence of λ on the applied force to the disk at different angles α: 1—$0^{\circ }$, 2—$45^{\circ }$, 3—$60^{\circ }$, 4—$90^{\circ }$.

**Figure 5:**
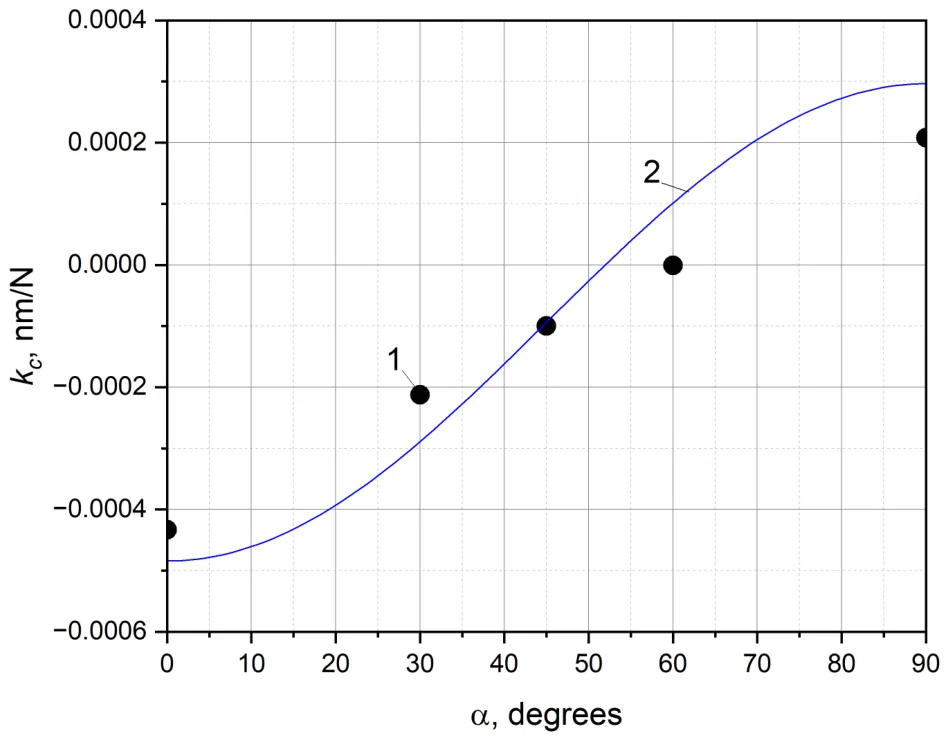
The dependence of the slope coefficient $k_{c}$ of straight lines Δλ(F) on the angle α: experiment (1) and calculation (2) according to Formula (19), taking into account the absolute values of deformation.

An example of the dependencies of Δλ(F) is shown in Figure 4. The approximation of the experimentally obtained values of Δλ(F) by a linear function gives the value $k_{c}$ of the slope of the straight line Δλ(F). The dependencies obtained of the coefficients $k_{c}$ on the angle α are shown in Figure 5. Figure 4 shows that the lines extrapolated at F=0 intersect the OY axis at different values of λ. Consequently, during the measurement of the dependencies Δλ(F), there was some change in the temperature of FBG.

From the data shown in Figure 4 and Figure 5, it follows that as the angle α increases, the resonant wavelength FBG always changes upward. At angles $\alpha <\alpha_{0}$, FBG is compressed and the resonant wavelength FBG decreases compared to the unstressed state, i.e., Δλ<0, and when the angle $\alpha_{0}$ is exceeded, FBG is stretched, respectively Δλ>0. The dependence $k_{c}\left(\alpha \right)$ was calculated according to Formula (19), taking into account the absolute values of the deformation (6) and (7) and the proportionality coefficient between the displacement of the resonant wavelength FBG and the relative deformation [5] $k_{B}=0.78\lambda_{B}$ nm. Young’s modulus was set to $3\times 10^{9}$ Pa. There is good agreement between the calculated dependence and the experimental data across the entire range of angles α.

The dependencies Δλ(α) and $k_{c}\left(\alpha \right)$ actually describe the same effect. However, the dependence of Δλ(α) is affected by the change in FBG temperature during measurements. The use of the coefficient $k_{c}\left(\alpha \right)$ eliminates the effect of temperature changes between measurements at different angles α, making this parameter more informative but less convenient to use than Δλ(α). It should also be noted that the dependencies shown in Figure 3 are given in relative values, and Figure 4 and Figure 5 show absolute values.

The dependence of Δλ(α) on static load was also measured without using a spectrometer, using the technique described above. The measurement result and the theoretical dependencies of Δλ(α) are shown in Figure 3.

The shape of the FBG reflection spectrum at different disk loads at different angles α did not differ. That is, the effects associated with induced birefringence were negligible in the case considered.

The object under study (disk with the glued fiber Bragg grating) was subjected to dynamic non-destructive tests. A freely falling steel cylinder with the parameters described above was used as an external impact. The cylinder reached velocity of $v={\left(2gh\right)}^{1/2}=1.7$ m/s at the moment of impact on the end of the PMMA disk. The longitudinal direction of FBG was at an angle α to the direction of incidence of the impact cylinder.

**Figure 6:**
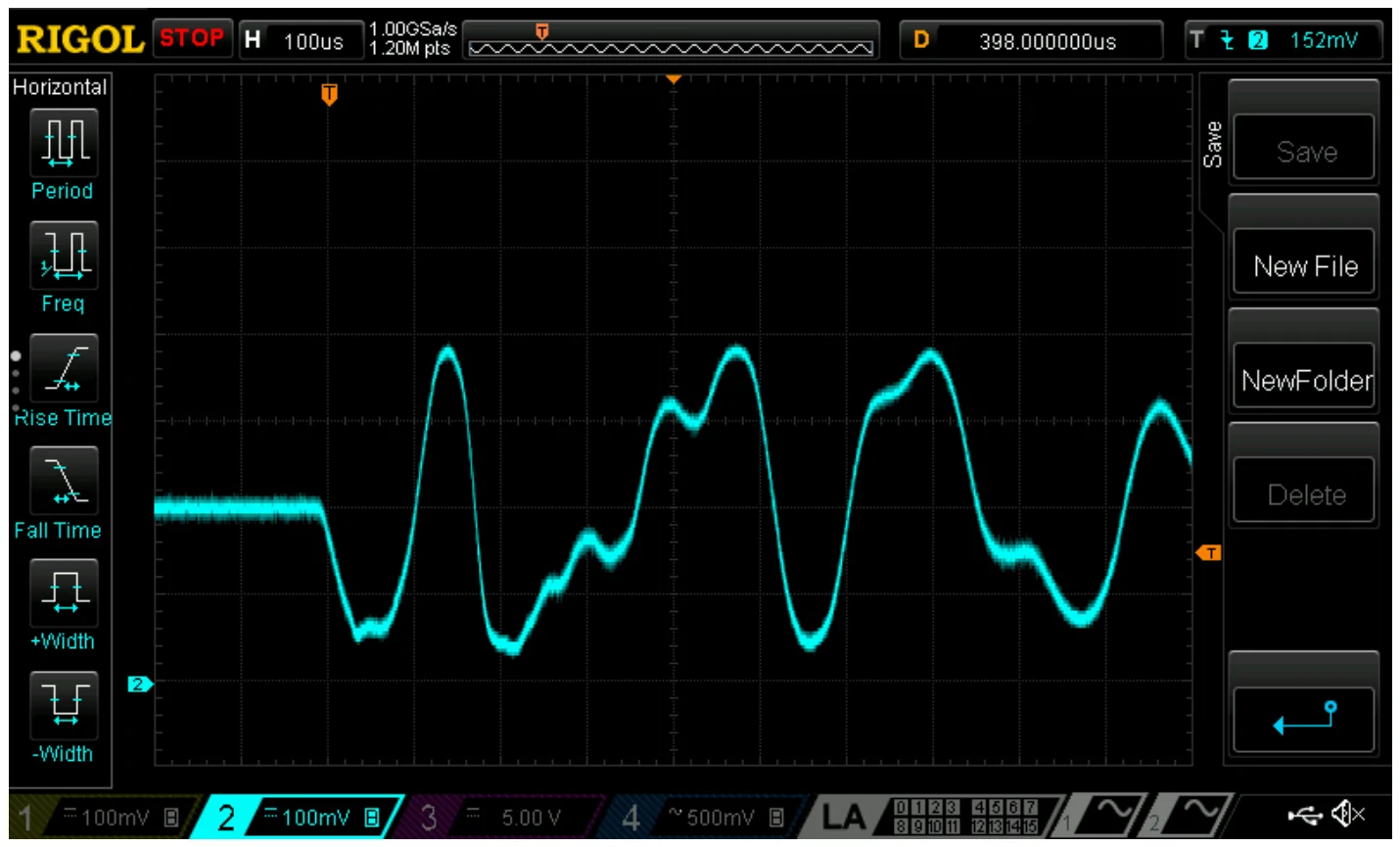
An example of waveform of the signal at α=0 under pulsed mechanical action.

A typical waveform of the signal at α=0 is shown in Figure 6. The operating point was chosen such that the photodetector signal value was approximately 1/2 of the maximum value (200 mV at the operating point, 400 mV is the maximum value). The reduction in signal corresponded to the compression of FBG [26] (Figure 6).

**Figure 7:**
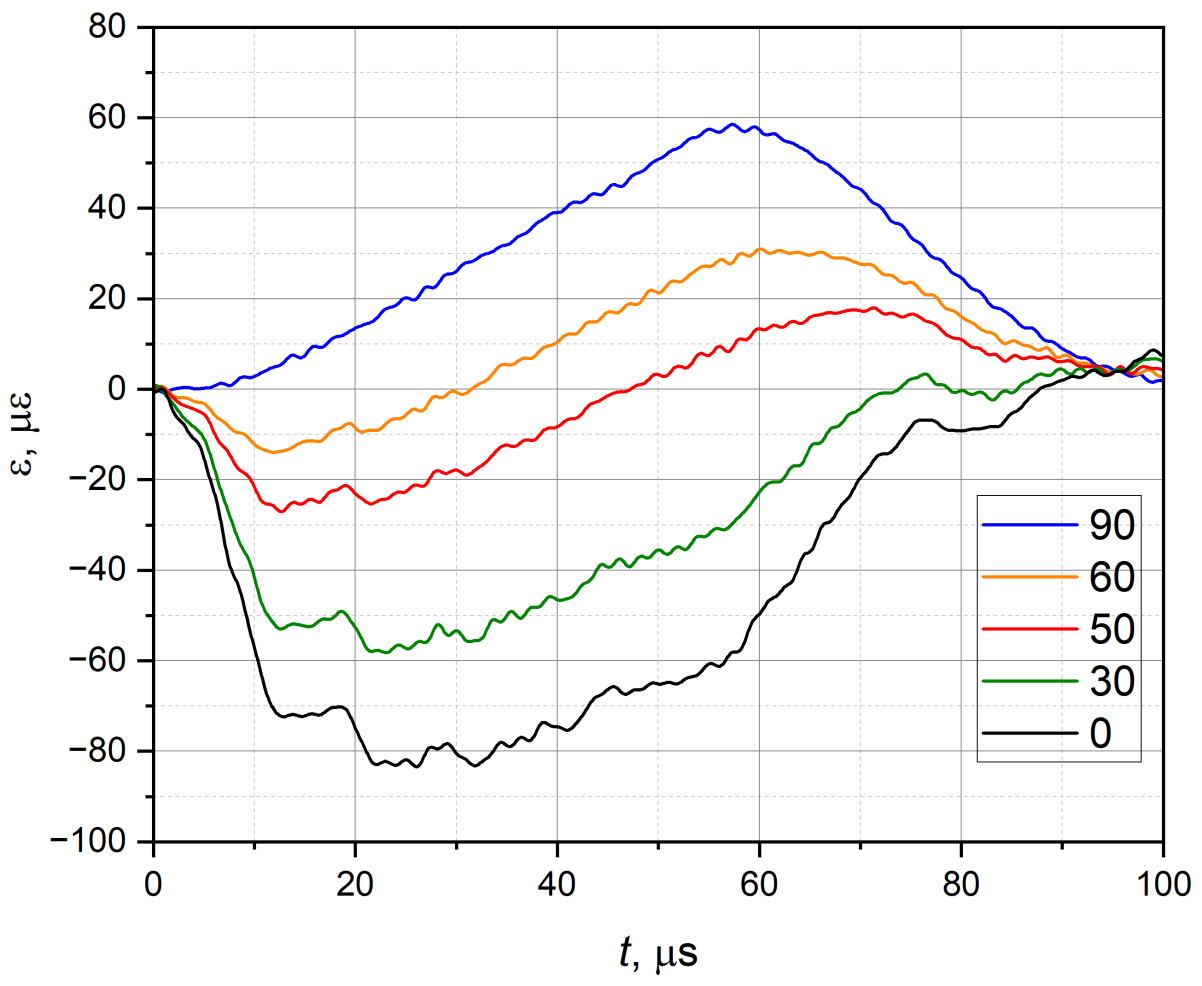
Time dependence of deformation along the FBG axis (numerical simulation) at different angles α: $0^{\circ }$, $30^{\circ }$, $50^{\circ }$, $60^{\circ }$, $90^{\circ }$.

**Figure 8:**
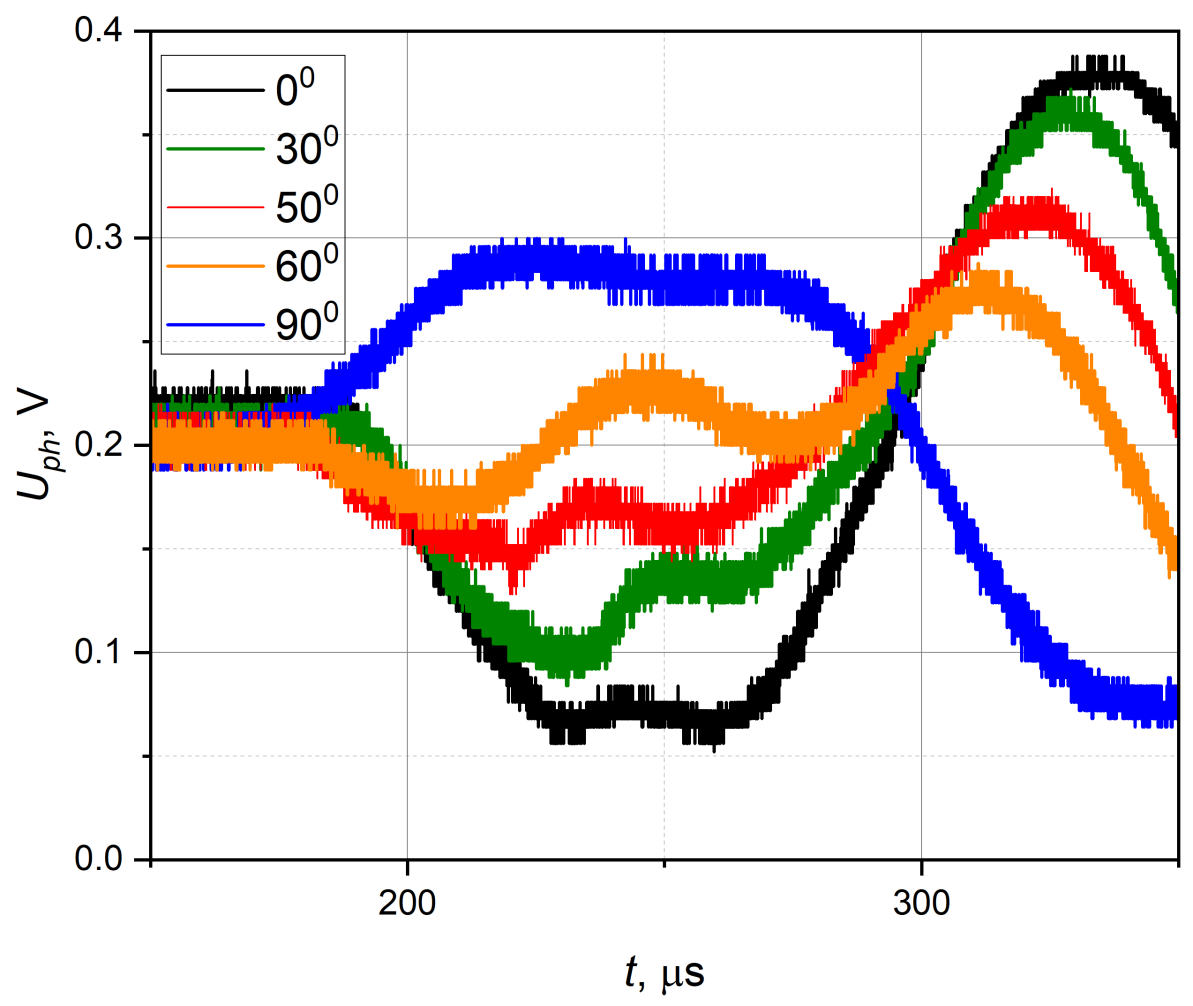
Waveforms of the signal from the photodetector (experiment) at various angles α: $0^{\circ }$, $30^{\circ }$, $50^{\circ }$, $60^{\circ }$, $90^{\circ }$.

The results of numerical simulation of the time dependence of the FBG deformation and the experimentally obtained waveforms are shown in Figure 7 and Figure 8. According to the calculation and experimental results, it can be seen that in the case of the FBG located along the incidence direction of the impact cylinder ($\alpha =0^{\circ }$), the relative deformations are negative, correspond to compression, and reach a minimum value relative to the FBG deformations located at other angles. When the FBG is positioned across the direction of incidence of the impact cylinder ($\alpha =90^{\circ }$), the relative deformations are positive, correspond to tension, and reach their maximum value relative to the deformations of FBGs located at other angles. In the case of α = 45–60°, the relative deformations change sign, the values of the relative deformation modulus are minimal with respect to the FBG deformation modules located at other angles. The least effect on the spectral response of FBG was observed at $\alpha =50^{\circ }$, which is close to the theoretical estimation (26) of the angle $\alpha_{0}$ under static exposure.

The ratio $r_{\epsilon }$ of the maximum value of the stretching wave to the minimum value of the compression wave, obtained by numerical modeling (Figure 7), is approximately 0.71, and the experimentally obtained (Figure 8) ratio is approximately 0.69.

## 5. Discussion

Formulas (11)–(19) describe the calculation of the FBG elongation, taking into account the deformation of the surrounding fiber medium along both axes (0X, 0Y), and the deformation along the 0Z axis is neglected in this model. Good agreement between theory and experimental data under static loading allows us to consider the accepted assumptions to be legitimate. The shape of the dependencies Δλ(α)/Δλ(α=0) and ϵ(α)/ϵ(α=0) coincides (Figure 3), as well as the dependence of $k_{c}\left(\alpha \right)$ on ϵ(α)/ϵ(α=0) in absolute values (Figure 5), but there is some difference at angles of $70^{\circ }\ldots 90^{\circ }$ (Figure 3), as well as the minimum and maximum values at the beginning (α≈0) and the end ($\alpha \approx 90^{\circ }$) of the angle range α. This presumably has the following explanation. In addition to the concentrated compressive force in the vertical position of the disk (Figure 1), the deformation of the FBG is affected by the distributed mass of the disk, equivalent to an increase in the compressive force under the influence of gravity. This may be the reason for the difference in dependencies shown in Figure 3. However, at a constant angle α, the additional constant compressive force does not affect the angle of inclination of the dependence Δλ(F). Therefore, it can be assumed that some difference between the calculated and experimental data (Figure 5) is due to the mechanical contact of the FBG with the environment, i.e., with the disk surface, leading to a change in the magnitude of the elastic-optical effect in the FBG, respectively, to a change in coefficient $k_{B}$. The value $k_{B}/\lambda_{B}$, assumed to be 0.78 in the calculation, corresponds to a free optical fiber, and a change in the effective elastic-optical constant leads to a change in the coefficient $k_{B}$.

Theoretically, the dependencies λ(F) (Figure 4) at F=0 should intersect at one point. However, as can be seen from the graphs, there is some difference from the idealized dependence. Thus, the values λ(F=0) of dependencies 1 and 2 (Figure 4) differ by 0.00623 nm, and 1 and 4 do not differ within the measurement error. The greatest difference between λ(F=0) was observed between dependencies 1 (or 4) and 3–0.0133 nm. Since the temperature coefficient FBG at a wavelength of 1310 nm is approximately 11.6 pm/°C, we obtain a temperature difference 0.38 °C and 0.8 °C, which is higher than the desired value required for accurate measurements. Therefore, it can be assumed that the measurement errors were caused by the FBG temperature change.

The ratio $r_{\epsilon }$ obtained by numerical simulation and experimentally (0.71 and 0.69) differs from the ratio $r_{\epsilon }$ for the static mode (0.612). It can be assumed that this difference is due to the need to take into account the dynamic properties of PMMA and the wave nature of the deformation change under pulsed action. Based on the dependencies obtained, it can be argued that the characteristic of the static load mode also appears under pulsed mechanical influences.

## 6. Conclusions

Good agreement between theoretical and experimental data on static loading of a disk with the fiber Bragg grating glued in the center at arbitrary angles between the FBG axis and the direction of force applied allows us to conclude that it is possible to use classical deformation theory and the fundamental properties of FBG to calculate the spectral response of the embedded FBG. It can be argued that this approach is also applicable to other solid isotropic media with embedded FBGs. The noted patterns—the dependence of the displacement of the resonant wavelength of an FBG on the angle between the FBG axis and the direction of force application, including the transition from compression to stretching, as well as the presence of an angle at which the FBG becomes insensitive to the force applied to the medium—will occur in any system, the linear dimensions of which are commensurate in two coordinates. On the basis of the dependencies obtained, it can be argued that all described properties also occur under pulsed mechanical influences.

## Data Availability

The original contributions presented in this study are included in the article. Further inquiries can be directed to the corresponding author.

## Abbreviations

The following abbreviations are used in this manuscript:

FBG	Fiber Bragg grating
PMMA	Polymethylmethacrylate
SL	Semiconductor laser
SM	Single mode
